# Concomitant Interstitial Lung Disease With Psoriasis

**DOI:** 10.7759/cureus.26979

**Published:** 2022-07-18

**Authors:** Prabin Kharibam, Sushma Laishram, Nikita Choudhary, Arnab Choudhury, Ravi Kant

**Affiliations:** 1 Department of Internal Medicine, All India Institute of Medical Sciences Rishikesh, Rishikesh, IND; 2 Department of Dermatology, Venereology, and Leprosy, All India Institute of Medical Sciences Rishikesh, Rishikesh, IND; 3 Department of Pathology, Sawai Man Singh Medical College, Jaipur, IND

**Keywords:** idiopathic pulmonary fibrosis (ipf), usual interstitial pneumonia (hip), non-specific interstitial lung disease (nsip), psoriasis, interstitial lung disease

## Abstract

Interstitial lung disease is occasionally reported in patients with psoriasis as drug-induced pneumonitis secondary to concomitant use of immunosuppressants in most cases. Although few cases have been reported describing the simultaneous existence of psoriasis and interstitial pneumonia, there are no reports that clearly show their direct association. A 55-year-old male known case of psoriasis and hypertension presented to the emergency department with complaints of pain and weakness of bilateral upper limbs following an episode of seizure and shortness of breath on exertion for one year. Following workup, the patient was diagnosed to have interstitial lung disease. There was no history of any immunosuppressant or use of biologics. So, immune dysfunction triggered by psoriasis might have caused the lung fibrotic changes. Careful monitoring of lung and skin lesions is vital for diagnosing psoriasis-associated pneumonia.

## Introduction

Psoriasis is a common chronic inflammatory skin disease characterized by epidermal hyperplasia and decreased epidermal turnover time [1]. Plaque psoriasis is the most common type, which commonly presents as erythematous, scaly, sharply demarcated, indurated plaques, particularly over the extensor surfaces and scalp [2].

Interstitial lung disease (ILD) is any lung disease affecting the parenchymal interstitial or loose-binding connective tissue. Most cases of ILD are reported in patients with psoriasis as drug-induced pneumonitis secondary to immunosuppressants and biologics [3,4]. Few cases have been reported describing the simultaneous existence of psoriasis and interstitial pneumonia [5]. However, their direct association is not clearly shown. We report a known case of psoriasis, which later developed into ILD.

## Case presentation

A 55-year-old male who is a bank employee by occupation from a lower-middle-class family presented to the emergency department with complaints of pain and weakness of bilateral upper limbs for one day, following an episode of seizure. The episode was sudden in onset and lasted for two minutes. It was associated with up rolling of the eyeball, urinary incontinence, and tongue bite followed by postictal confusion, which lasted for around six hours. Upon regaining consciousness, the patient developed bilateral upper limb weakness associated with a restricted range of movements. There was no history of a similar episode in the past. The patient also complained of shortness of breath on exertion for the past year. It was insidious in onset, progressive, and not associated with chest pain, palpitation, pedal edema, or decreased urine output. He was treated in the line of bronchial asthma outside, and he had been using a salbutamol inhaler for the same. There was no history suggestive of orthopnea or paroxysmal nocturnal dyspnea. There was no history of cough with hemoptysis. The patient was a known case of psoriasis for 15 years. The lesions initially started in the scalp and later involved the trunk and extremities. He had been using only topical steroids and oral antihistamines on and off for the past 15 years as his body surface area (BSA) involved was less, which worked for him. There was no history of methotrexate or immunosuppressant use for his psoriasis at any point in time. He was a known case of hypertension for six years on regular medication (tablet telmisartan 40 mg once daily). He was a non-smoker and occasional drinker.

**Figure 1:**
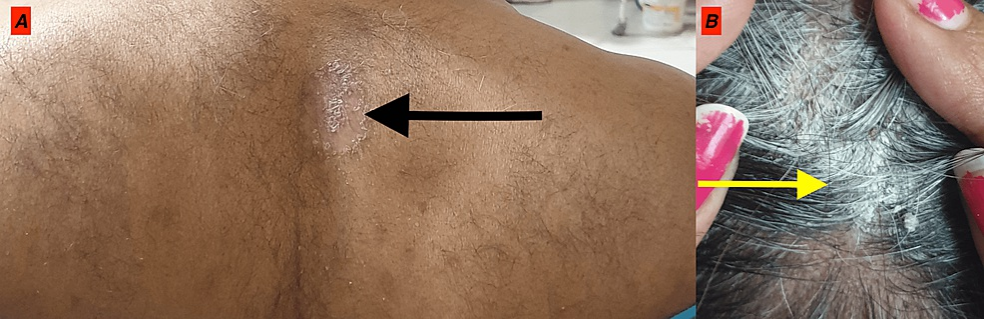
(A) A well-defined scaly plaque of psoriasis and (B) well-defined thick adherent silvery scales in the scalp

On examination, he was conscious, cooperative, and oriented to time, place, and person. The vitals were stable. Basal oxygen saturation was over 98%. Physical examination revealed well-demarcated erythematous plaques with scales over the upper back and the extensors of bilateral legs and a few discrete scaly plaques on the scalp with a BSA of 4% and psoriasis area and severity index (PASI) score of 2.3 (Figure 1).

**Figure 2:**
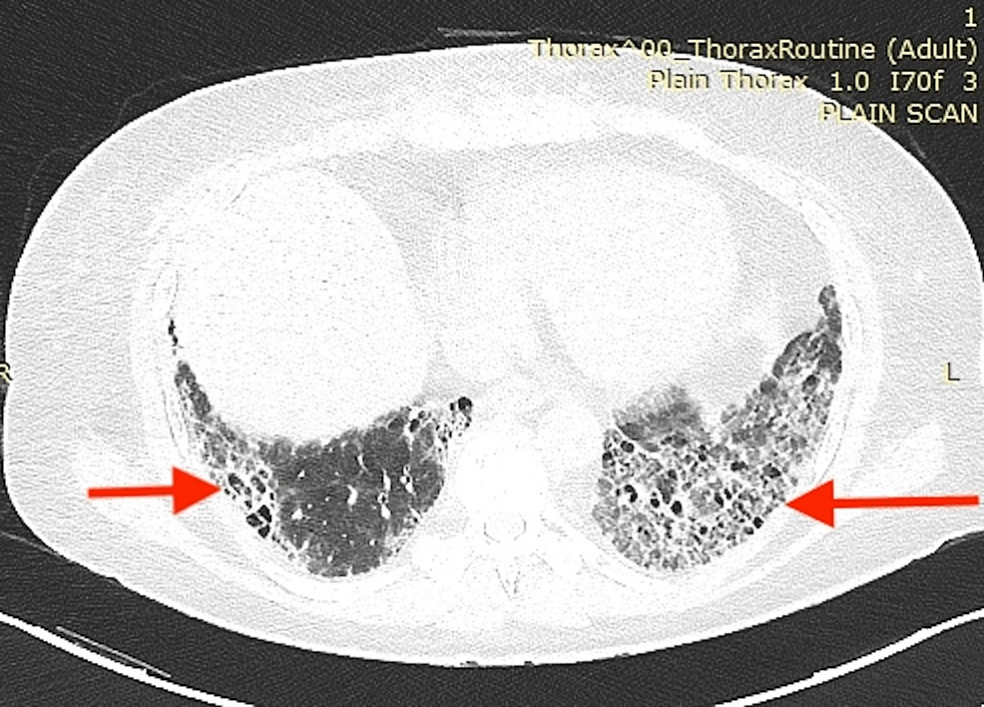
HRCT thorax showing heterogenous subpleural and basal predominant honeycombing with traction bronchiectasis in the bilateral lung HRCT: High-resolution computed tomography.

No evidence of joint inflammation or deformity was noted on the musculoskeletal exam. However, tenderness was present in bilateral shoulder joints with the muscle power of 4/5 in each arm. Auscultation revealed fine crepitations in the bilateral chest and interscapular areas. Other systemic examinations were non-significant. ECG was normal. An echocardiogram showed a structurally normal heart without evidence of valve disease. The pulmonary function test was suggestive of severe restrictive lung disease and diffusion defect. High-resolution computed tomography (HRCT) thorax was obtained, suggesting an ILD - usual interstitial pneumonia (UIP) pattern (Figure 2).

**Figure 3:**
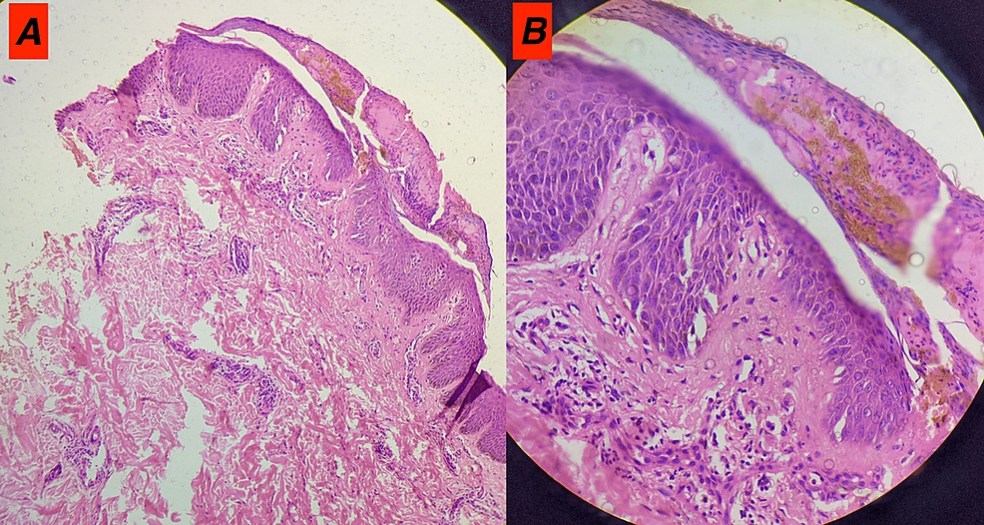
(A) 100X magnification, H&E section shows acanthosis, parakeratosis with neutrophils, and thinning of suprapapillary plates. (B) 400X magnification, H&E section shows parakeratosis with neutrophils and perivascular lymphocytic infiltrate. H&E: Hematoxylin and eosin stain.

An extensive review of history was done again to elicit a possible history of exposure to substances (occupational and iatrogenic) causing ILD, which was negative. There was no family history of ILD. Extensive workup for connective tissue disorder yielded negative results. Histopathological examination of skin biopsy showed downward elongation of rete ridges, parakeratosis, and alternating hypo- and hypergranulosis in the epidermis. Neutrophilic collection in parakeratotic stratum corneum (Munro microabscess) was also seen. There was thinning of suprapapillary epidermis along with elongation of dermal papillae. The upper and mid dermis showed lymphocytic infiltrate around blood vessels. The biopsy findings were consistent with features of psoriasis (Figure 3).

Initially, symptomatic treatment was given. He was started on injection phenytoin 100 mg total dissolved solids (TDS) for seizure and was given nebulization with budesonide and duolin (levosalbutamol + ipratropium bromide) for shortness of breath. He improved symptomatically. Dislocated shoulders were reduced and managed conservatively. He was discharged on oral anti-epileptics and metered dose inhaler (MDI) duolin. Oral antihistamines and topical steroids and emollients were given for psoriasis. The patient was advised to enroll for a lung transplant but was lost to follow-up.

## Discussion

Psoriasis is a chronic inflammatory skin disease with systemic effects associated with several comorbidities, including cardiometabolic diseases [1,5]. The risk factors include genetic susceptibility and environmental triggers such as streptococcal infection, stress, smoking, obesity, and alcohol consumption [1]. It is associated with systemic manifestations in many organ systems. Psoriatic arthritis occurs in 40% of patients with moderate to severe psoriasis, characterized by involvement of the distal interphalangeal joints of the hands and feet and the absence of rheumatoid factor [2].

Psoriasis-related immune dysfunction may cause an abnormal immunologic response in the lung parenchyma. It is suggested that immune dysfunction in patients with psoriasis might also cause inflammation and fibrotic process in the lung interstitium. Type 17 helper T cells, also the hallmark of pathogenesis in psoriasis, were reported to be one of the common pathways contributing to alveolitis. Activated T(H)17 cells produce several mediators such as interleukin 17A, 17F, and 22, which induce keratinocyte proliferation. Moreover, IL-17 contributes to alveolitis and enhances cytokine production of pulmonary fibroblasts. Ishikawa et al. have shown that dermal cell proliferation in psoriasis is due to activated transforming growth factor (TGF)-α, which may also be associated with activated TGF-β, leading to pulmonary fibrosis. In their case series study, the clinical and radiographic characteristics of patients with concomitant psoriasis and ILD were reviewed. Of 447 patients, 21 ILD patients (4.7%) had concomitant psoriasis or psoriatic arthritis, with 63.6% not being previously or concomitantly exposed to immunosuppressants and 11 (52.4%) patients with a clinical diagnosis of idiopathic pulmonary fibrosis (IPF). Regarding radiographic findings, the UIP pattern was more common (42.9%) than the nonspecific interstitial pneumonia/organizing pneumonia (NSIP/OP) pattern [3].

In a study by Kawamoto et al. in Japan, of the 392 patients with psoriasis, interstitial pneumonia was found in eight patients (2%) with bilateral ground-glass and/or irregular linear (reticular) opacity in the lower lung zone as the most common in CT findings. It has been suggested that IL-23/IL-17 may be responsible for interstitial pneumonia in psoriasis [4]. Also, increasing numbers of ILD associated with biologics have been reported. Many were associated with TNF-α inhibitors. In a study by Matsumoto et al., which reviewed 246 psoriasis patients treated with biological agents, pulmonary adverse events were seen in 22 cases, of which 11 cases were diagnosed as drug-induced ILD (mainly TNF-α inhibitor) [5].

In our patient, there is no history of previous exposure to immunosuppressants or biologics in the past. He was a non-smoker. So, it is possible that immune dysfunction in the lungs triggered by psoriasis might cause fibrotic change. There is no history of other connective tissue diseases that may cause interstitial pneumonia. However, further study on a large population is required to prove a causal association between psoriasis and interstitial pneumonia. Also, he was misdiagnosed with a case of bronchial asthma for breathlessness. So, spirometry can be advised as a screening test, and lung lesions in psoriasis patients should be more extensively investigated using CT in more extensive studies. Careful monitoring of lung and skin lesions is essential for diagnosing psoriasis-associated pneumonia.

## Conclusions

A patient with psoriasis under biologics treatment has a higher risk of developing drug-induced ILD. Nevertheless, patients with psoriasis not on biologics treatment may rarely develop ILD concomitantly due to abnormal immune dysregulation inducing pulmonary fibrosis. A high index of suspicion is needed to diagnose concomitant psoriasis and ILD. Patients with psoriasis should be screened for ILD with spirometry, even those who are not on biologics treatment.
